# Four cases of cytokine storm after COVID-19 vaccination: Case report

**DOI:** 10.3389/fimmu.2022.967226

**Published:** 2022-08-15

**Authors:** Kazuhiro Murata, Naoki Nakao, Naoki Ishiuchi, Takafumi Fukui, Narutaka Katsuya, Wataru Fukumoto, Hiroko Oka, Naotaka Yoshikawa, Takafumi Nagao, Akira Namera, Naoya Kakimoto, Naohide Oue, Kazuo Awai, Kanji Yoshimoto, Masataka Nagao

**Affiliations:** ^1^ Center for Cause of Death Investigation Research Graduate School of Biomedical and Health Sciences, Hiroshima University, Hiroshima, Japan; ^2^ Department of Forensic Medicine, Graduate School of Biomedical and Health Sciences, Hiroshima University, Hiroshima, Japan; ^3^ Department of Molecular Pathology, Graduate School of Biomedical and Health Sciences, Hiroshima University, Hiroshima, Japan; ^4^ Department of Oral and Maxillofacial Radiology, Graduate School of Biomedical and Health Sciences, Hiroshima University, Hiroshima, Japan; ^5^ Department of Diagnostic Radiology, Graduate School of Biomedical and Health Sciences, Hiroshima University, Hiroshima, Japan; ^6^ Department of Food Sciences and Biotechnology, Graduate School of Science and Technology, Hiroshima Institute of Technology, Hiroshima, Japan

**Keywords:** COVID-19, vaccination, RNA sequencing, immunology, side effects

## Abstract

The global coronavirus disease 2019 (COVID-19) pandemic has led to the rapid development of vaccines against this disease. Despite the success of the international vaccination program, adverse events following vaccination, and the mechanisms behind them, remain poorly understood. Here we present four cases of death following receipt of a second dose of COVID-19 vaccine, with no obvious cause identified at autopsy. Using RNA sequencing, we identified genes that were differentially expressed between our post-vaccination cases and a control group that died of blood loss and strangulation. Three hundred and ninety genes were found to be upregulated and 115 genes were downregulated in post-vaccination cases compared with controls. Importantly, genes involved in neutrophil degranulation and cytokine signaling were upregulated. Our results suggest that immune dysregulation occurred following vaccination. Careful observation and care may be necessary if an abnormally high fever exceeding 40°C occurs after vaccination, even with antipyretic drugs.

## Introduction

Coronavirus disease 2019 (COVID-19) has spread rapidly worldwide, with the number of infected cases exceeding approximately 550 million as of July 2022, defining it as a significant global health concern [WHO coronavirus (COVID-19) dashboard. Geneva: World Health Organization, https://covid19.who.int/table]. While there is growing interest in the use of vaccination for reducing disease severity, there are some reports of vaccination-related side effects, such as immune thrombocytopenia, myocarditis, and death (1–3). Meanwhile several studies have reported systemic immune response syndrome (SIRS) after vaccination (4, 5). We experienced four cases who died 1–10 days after receiving their second COVID-19 vaccination. Their body temperatures were estimated to be abnormally high at the time of death. Autopsies were performed on these patients but revealed no information about the cause of death, with evidence that pathologic analysis showed no changes to the primary organs. Here, we report the cause of death estimated by sequencing RNA.

## Materials and methods

### Case profiles

The profiles of four cases who died at home after receiving the COVID-19 vaccine are summarized in **Table 1**. Three cases received two doses of the Moderna mRNA COVID-19 vaccine, tozinameran, and one case received two doses of the Pfizer-BioNTech mRNA COVID-19 vaccine, elasomeran. The time from receipt of the second dose to death was 1–10 days. In case 3, side effects following vaccination, such as fever and headache, were not observed on the day before death, whereas in the other cases, they were observed on the day before death. Autopsies revealed no information about the cause of death for any patient, and pathologic analysis showed findings of sudden death, such as congestion of primary organs, and no information about the cause of death, including myocarditis. The postmortem interval inferred from postmortem phenomena and coroner’s rectal temperature measurements estimated high body temperatures for all cases at the time of death (6). Two female cases who experienced sudden death by blood loss (54 years old) and strangulation (86 years old) and had also received two doses of tozinameran were used as controls for RNA sequencing analysis.

**Table 1:** Profiles of investigated 4 fatalities and 2 controls.

	Case 1	Case 2	Case 3	Case 4	Control 1	Control 2
Age (years)	30	52	23	31	58	86
Gender	Male	Male	Male	Male	Female	Female
Season	Summer	Autumn	Autumn	Winter	Summer	Summer
Height (cm)	169	174	168	168	149	149
Weight (kg)	67.4	89.5	58.9	62.7	70	45
Postmortem interval (hours)	6〜8	20	6〜8	7〜8	6	7
Rectal temperature in coroner’s inspection (℃)	37	33	33	36	33	33
Time interval between vaccination and death (days)	2	3	10	1	16	94
Body temperature* (℃)	41〜43	42〜46	39〜41	43〜44	36.2	37.9
Vaccine	Tozinameran	Tozinameran	Elasomeran	Tozinameran	Tozinameran	Tozinameran
Used antipyretic drug	Acetaminophen	No information	No information	Loxoprofen Sodium	No information	No information

^*^Body temperature: estimated body temperature at the time of death Figure legends.

### RNA extraction and sequencing

RNA extraction from blood samples was performed using ISOGEN-LS (NIPPON GENE, Japan) following the manufacturer’s protocols. Transcriptome profiling using next-generation sequencing was conducted by Azenta Japan Corp (formerly GENEWIZ, Japan). In brief, total RNA was quantified and qualified by Qubit RNA assay (Thermo Fisher Scientific, USA) and TapeStation RNA ScreenTape (Agilent Technologies, USA). Total RNA (400 ng) was treated using an NEBNext rRNA depletion kit (Human/Mouse/Rat) (NEB, USA) to eliminate rRNA and prepare the cDNA library. Synthesis of cDNA followed by transcriptome library preparation was conducted using the MGIEasy RNA directional library prep kit v2.0 (MGI tech, China), in which dUTP was incorporated in second-strand cDNA synthesis instead of dTTP, to block PCR amplification against the second-strand templates and enable strand-specific transcriptome profiling. A 12-cycle PCR amplification was performed to increase library yield. The resulting transcriptome sequencing libraries were quantified by Qubit DNA Assay (Thermo Fisher Scientific) and their fragment size distribution was confirmed by TapeStation D1000 ScreenTape (Agilent). The adapter sequences used in the library preparation were:

forward: AAGTCGGAGGCCAAGCGGTCTTAGGAAGACAA;

reverse: AAGTCGGATCGTAGCCATGTCGTTCTGTGAGCCAAGGAGTTG.

The resulting double-stranded library fragments were pooled/multiplexed in equimolar amounts and further processed into single-stranded circular DNA (sscDNA), which was the final form of the MGI library. The sscDNA libraries were quantified with the Qubit ssDNA assay kit (Thermo Fisher Scientific) and used to generate DNA nanoballs (DNBs) by rolling circle replication reaction. DNBs were then loaded into a flow cell for sequencing on a DNBSEQ-G400 platform (MGI tech) with 150-bp PE configuration, in accordance with the manufacturer’s instructions. RNA sequencing data reported are available in the DDBJ Sequenced Read Archive under the accession numbers DRA013514.

### Bioinformatics

Quality control for the RNA sequencing raw reads were performed with FASTQC v0.11.9, and trimming was done with Trimmomatic v0.39. Read counts were estimated by using Kallisto v0.46.0 with default settings (7). Kallisto is a program for quantifying abundances of transcripts from RNA sequencing data, requiring a reference transcriptome index for abundance estimation. We got the reference transcriptome data from GENCODE Human Release 39 (GRCh38.p13). Estimated abundance of transcripts were summarized to the gene level with Tximport v1.18.0. Differential gene expression analysis was performed using DESeq2 v1.30.1, which provides methods for differential expression testing by employing a negative binomial distribution and a shrinkage estimator for the distribution’s variance (8). P-values were calculated using a Wald test provided within DESeq2. Genes were identified as significantly differentially expressed genes if they showed a minimum log2 fold change ±1.0 and had a p-value <0.01.

### Pathway analysis

The GO annotation and KEGG pathway analyses were performed by using the Metascape online tool (http://metascape.org/gp/index.html#/main/step1) pathway analysis (9).

## Results

**Figure 1:**
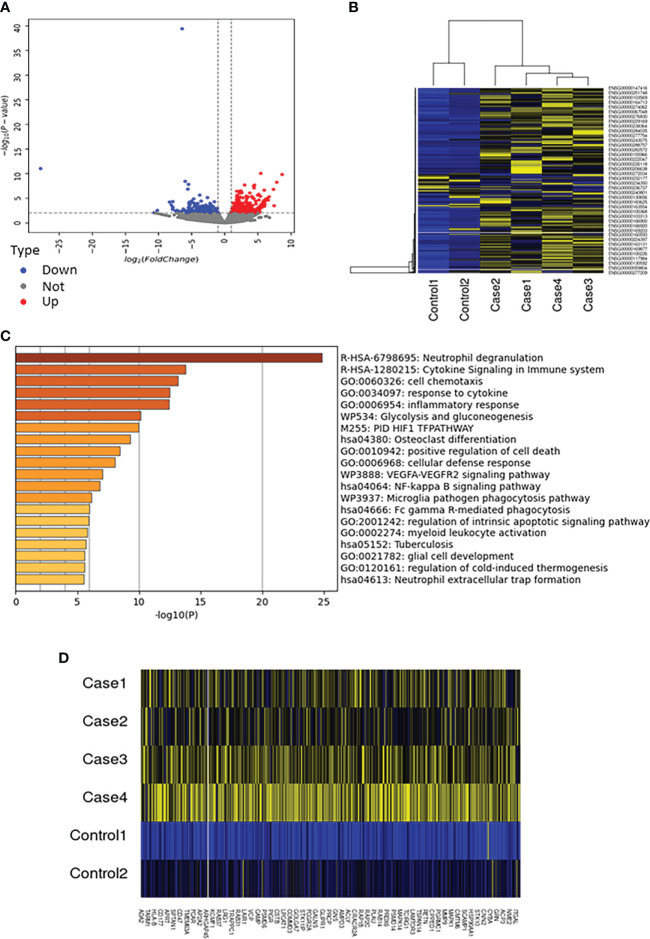
Genes associated with neutrophil degranulation and cytokine signaling were significantly elevated in the unknown group. **(A)** Volcano plots showing significantly different expressions of genes between the unknown and control groups (p<0.01). Red points, upregulated differentially expressed genes; blue points, downregulated differentially expressed genes; gray points, genes with no significant differences in expression. **(B)** Heatmap of different expressions of genes between the unknown and control groups (p<0.01). Yellow represents upregulation and blue represents downregulation. **(C)** The GO terms and KEGG pathways enrichment analysis for significantly upregulated genes between the unknown and control groups (p<0.01). **(D)** Heatmap of genes involved in neutrophil degranulation between the unknown and control groups. Yellow represents upregulation and blue represents downregulation.

The cause of death was estimated by sequencing RNA obtained from peripheral blood. We obtained an average of 39.1 million 150-base pair (bp) paired-end (PE) reads per sample. Of the 43,751 genes with nonzero total read counts, there were 505 significantly differentially expressed genes between individuals with unknown cause of death (unknown group) and the control group. Volcano plots showed a clear difference in mRNA expression between the unknown and control groups (**Figure 1A**). On the basis of the screening criteria, 390 genes were upregulated and 115 genes were downregulated (log2 fold change ≧ ± 1, p<0.01) (**Supplementary Tables 1**, **2**). Hierarchical clustering revealed clear differences in mRNA expression between the unknown and control groups (**Figure 1B**). Gene ontology (GO) biological processes terms and Kyoto encyclopedia of genes and genomes (KEGG) pathway enrichment analysis estimated that genes associated with neutrophil degranulation were markedly higher in the unknown group than in the control group (**Figure 1C**). Particular factors associated with neutrophil degranulation were increased, including PLAU (18.85-fold), CEACAM3 (6.58-fold), and FCGR3B (6.26-fold), and several genes involved in cytokine production and signaling were also elevated in the unknown group (**Figure 1D**). Together, these results provide evidence of a hyperactive immune response in the group with unknown cause of death.

## Discussion

Although COVID-19 infection has spread worldwide, protection through vaccination has been clearly effective in many countries. However, many drugs and vaccines have side effects, and. COVID-19 vaccines have been associated with several reports of adverse events including death (10–12). Thus, it is important to gather information about the risk of vaccination, although it can be difficult to identify the cause of death without clear data. While the causes of death of the cases reviewed in this study could not be identified by autopsy, RNA sequencing of blood samples obtained and properly stored shortly after death provided valuable information. RNA sequencing analysis using postmortem specimens has rarely been performed because of the degradation of RNA due to postmortem changes. However, in the present case group, it was possible to collect blood samples within 24 hours after death, which was relatively early, and RNA sequencing analysis may have been successful (13).

Although we have no way of knowing whether the cases we reported met these criteria, the RNA sequencing results suggested that an abnormal secretion of cytokines, possibly a cytokine storm, may have occurred after vaccination, resulting in SIRS and death. SIRS is defined as fulfilling at least two of the following criteria: fever >38.0°C or hypothermia <36.0°C, tachycardia >90 beats/minute, tachypnea >20 breaths/minute, leukocytosis >12×10^9^/L or leucopoenia <4×10^9^/L (14). SIRS is induced by various factors, such as infection, trauma, surgery, and ischemia. It has been reported that SIRS can occur due to COVID-19 infection (15). COVID-19 vaccination corresponds to pseudo-COVID-19 infection. Therefore, vaccination may induce SIRS. In the present four victims, it is assumed that the immune function was sensitized by the first vaccination and that the second vaccination made the patients more susceptible to developing SIRS. Meanwhile, it is possible that these victims had a constitutional predisposition to be more prone to develop SIRS due to vaccination. However, our results do not indicate what mainly caused this aberrant cytokine response. Further studies such as analysis of single nucleotide polymorphisms are needed. It is important to note that vaccination remains essential to prevent the spread of infection and should not be considered dangerous on the basis of these cases alone. Additional research is needed to identify risk factors responsible for severe adverse events associated with vaccination.

## Conclusions

We present four cases of death following receipt of a second dose of COVID-19 vaccine, with no obvious cause identified at autopsy. RNA sequencing revealed that genes involved in neutrophil degranulation and cytokine signaling were upregulated in these cases, suggesting that immune dysregulation occurred following vaccination. Careful observation and care may be necessary if an abnormally high fever exceeding 40°C occurs after vaccination, even with antipyretic drugs.

## Data availability statement

The data presented in the study are deposited in the DDBJ Sequenced Read Archive repository, accession number DRA013514.

## Ethics statement

Ethical review and approval was not required for the study on human subjects in accordance with the local legislation and institutional requirements. Written informed consent for the publication of any potentially identifiable data or images was not required for this study in accordance with the national legislation and the institutional requirements.

## Author contributions

KM and NN performed the majority of the experiments and data analysis. NN, NI, TF, NaK, WF, HO, NY, TN, AN, NaoK, NO, KA, and MN provided data samples. NY, AN, and KY helped to write the paper. KM, NN, NI, and MN conceived of the project, planned and wrote the paper. All authors contributed to the article and approved the submitted version.

## Acknowledgments

We thank Edanz (https://jp.edanz.com/ac) for editing a draft of this manuscript. This work was carried out at the Analysis Center of Life Science, Natural Science Center for Basic Research and Development, Hiroshima University.

## Conflict of interest

The authors declare that the research was conducted in the absence of any commercial or financial relationships that could be construed as a potential conflict of interest.

## Publisher’s note

All claims expressed in this article are solely those of the authors and do not necessarily represent those of their affiliated organizations, or those of the publisher, the editors and the reviewers. Any product that may be evaluated in this article, or claim that may be made by its manufacturer, is not guaranteed or endorsed by the publisher.
